# Supplementary material for: SpaLSTF: Diffusion-based generative model with BiLSTM and XCA-Transformer for spatial transcriptomics imputation
Source: PLoS Comput Biol. 2026 Feb 10;22(2):e1013954. doi: 10.1371/journal.pcbi.1013954 (PMC12912704; doi:10.1371/journal.pcbi.1013954)
Supplement: S1 Table — (DOCX) [file pcbi.1013954.s001.docx]

**S1Table. Cluster results of all methods in all datasets.**

| Datasets | Methods | ARI | AMI | Homo | NMI |
| --- | --- | --- | --- | --- | --- |
| Dataset1 | Tangram | 0.135 | 0.235 | 0.252 | 0.239 |
|  | gimVI | 0.072 | 0.154 | 0.177 | 0.159 |
|  | SpaGE | 0.147 | 0.245 | 0.277 | 0.250 |
|  | stPlus | 0.202 | 0.321 | 0.366 | 0.326 |
|  | uniport | 0.0535 | 0.099 | 0.116 | 0.105 |
|  | spscope | 0.048 | 0.091 | 0.108 | 0.097 |
|  | diffusion | 0.152 | 0.273 | 0.306 | 0.277 |
|  | SpaLSTF | 0.251 | 0.320 | 0.340 | 0.324 |
| Dataset2 | Tangram | 0.196 | 0.324 | 0.355 | 0.343 |
|  | gimVI | 0.373 | 0.514 | 0.555 | 0.529 |
|  | SpaGE | 0.530 | 0.689 | 0.754 | 0.701 |
|  | stPlus | 0.434 | 0.614 | 0.668 | 0.628 |
|  | uniport | 0.364 | 0.508 | 0.557 | 0.523 |
|  | spscope | 0.490 | 0.631 | 0.673 | 0.644 |
|  | diffusion | 0.538 | 0.700 | 0.754 | 0.709 |
|  | SpaLSTF | 0.593 | 0.735 | 0.789 | 0.743 |
| Dataset3 | Tangram | 0.171 | 0.262 | 0.273 | 0.269 |
|  | gimVI | 0.469 | 0.570 | 0.613 | 0.575 |
|  | SpaGE | 0.346 | 0.491 | 0.538 | 0.498 |
|  | stPlus | 0.398 | 0.517 | 0.548 | 0.523 |
|  | uniport | 0.472 | 0.539 | 0.536 | 0.543 |
|  | spscope | 0.286 | 0.437 | 0.474 | 0.446 |
|  | diffusion | 0.548 | 0.584 | 0.558 | 0.586 |
|  | SpaLSTF | 0.571 | 0.595 | 0.581 | 0.598 |
| Dataset4 | Tangram | 0.062 | 0.126 | 0.159 | 0.132 |
|  | gimVI | 0.118 | 0.235 | 0.268 | 0.239 |
|  | SpaGE | 0.056 | 0.111 | 0.141 | 0.117 |
|  | stPlus | 0.084 | 0.172 | 0.201 | 0.176 |
|  | uniport | 0.201 | 0.314 | 0.306 | 0.315 |
|  | spscope | 0.085 | 0.166 | 0.190 | 0.170 |
|  | diffusion | 0.277 | 0.333 | 0.329 | 0.334 |
|  | SpaLSTF | 0.252 | 0.346 | 0.349 | 0.347 |
| Dataset5 | Tangram | 0.325 | 0.619 | 0.618 | 0.629 |
|  | gimVI | 0.448 | 0.686 | 0.688 | 0.693 |
|  | SpaGE | 0.485 | 0.727 | 0.740 | 0.735 |
|  | stPlus | 0.495 | 0.732 | 0.746 | 0.739 |
|  | uniport | 0.309 | 0.569 | 0.537 | 0.576 |
|  | spscope | 0.367 | 0.618 | 0.610 | 0.628 |
|  | diffusion | 0.620 | 0.796 | 0.796 | 0.801 |
|  | SpaLSTF | 0.647 | 0.800 | 0.802 | 0.805 |
| Dataset6 | Tangram | 0.109 | 0.264 | 0.315 | 0.269 |
|  | gimVI | 0.199 | 0.408 | 0.505 | 0.411 |
|  | SpaGE | 0.175 | 0.315 | 0.395 | 0.319 |
|  | stPlus | 0.266 | 0.410 | 0.487 | 0.412 |
|  | uniport | 0.232 | 0.356 | 0.368 | 0.357 |
|  | spscope | 0.216 | 0.364 | 0.427 | 0.368 |
|  | diffusion | 0.292 | 0.456 | 0.492 | 0.458 |
|  | SpaLSTF | 0.334 | 0.477 | 0.512 | 0.478 |
| Dataset7 | Tangram | 0.311 | 0.516 | 0.499 | 0.520 |
|  | gimVI | 0.618 | 0.760 | 0.785 | 0.763 |
|  | SpaGE | 0.712 | 0.839 | 0.897 | 0.841 |
|  | stPlus | 0.612 | 0.791 | 0.852 | 0.794 |
|  | uniport | 0.872 | 0.900 | 0.888 | 0.901 |
|  | spscope | 0.618 | 0.760 | 0.785 | 0.763 |
|  | diffusion | 0.896 | 0.908 | 0.908 | 0.909 |
|  | SpaLSTF | 0.868 | 0.895 | 0.897 | 0.896 |
| Dataset8 | Tangram | 0.163 | 0.192 | 0.243 | 0.233 |
|  | gimVI | 0.243 | 0.349 | 0.397 | 0.382 |
|  | SpaGE | 0.024 | 0.027 | 0.059 | 0.062 |
|  | stPlus | 0.289 | 0.354 | 0.354 | 0.377 |
|  | uniport | 0.097 | 0.136 | 0.159 | 0.168 |
|  | spscope | 0.015 | 0.029 | 0.061 | 0.063 |
|  | diffusion | 0.077 | 0.126 | 0.176 | 0.172 |
|  | SpaLSTF | 0.104 | 0.171 | 0.223 | 0.214 |
| Dataset9 | Tangram | 0.165 | 0.319 | 0.335 | 0.325 |
|  | gimVI | 0.310 | 0.474 | 0.529 | 0.480 |
|  | SpaGE | 0.370 | 0.529 | 0.599 | 0.535 |
|  | stPlus | 0.244 | 0.415 | 0.470 | 0.422 |
|  | uniport | 0.376 | 0.510 | 0.531 | 0.515 |
|  | spscope | 0.228 | 0.395 | 0.445 | 0.406 |
|  | diffusion | 0.191 | 0.377 | 0.393 | 0.384 |
|  | SpaLSTF | 0.230 | 0.421 | 0.441 | 0.427 |
| Dataset10 | Tangram | 0.144 | 0.207 | 0.245 | 0.216 |
|  | gimVI | 0.283 | 0.374 | 0.410 | 0.380 |
|  | SpaGE | 0.178 | 0.237 | 0.260 | 0.244 |
|  | stPlus | 0.220 | 0.313 | 0.338 | 0.319 |
|  | uniport | 0.094 | 0.132 | 0.151 | 0.140 |
|  | spscope | 0.055 | 0.130 | 0.208 | 0.151 |
|  | diffusion | 0.131 | 0.212 | 0.230 | 0.218 |
|  | SpaLSTF | 0.152 | 0.240 | 0.289 | 0.250 |
| Dataset11 | Tangram | 0.131 | 0.205 | 0.244 | 0.213 |
|  | gimVI | 0.149 | 0.240 | 0.276 | 0.247 |
|  | SpaGE | 0.328 | 0.407 | 0.480 | 0.414 |
|  | stPlus | 0.387 | 0.536 | 0.637 | 0.542 |
|  | uniport | 0.131 | 0.198 | 0.229 | 0.205 |
|  | spscope | 0.275 | 0.356 | 0.402 | 0.362 |
|  | diffusion | 0.287 | 0.369 | 0.408 | 0.375 |
|  | SpaLSTF | 0.294 | 0.351 | 0.386 | 0.357 |
| Dataset12 | Tangram | 0.088 | 0.139 | 0.160 | 0.154 |
|  | gimVI | 0.220 | 0.353 | 0.413 | 0.368 |
|  | SpaGE | 0.117 | 0.226 | 0.264 | 0.242 |
|  | stPlus | 0.140 | 0.230 | 0.231 | 0.240 |
|  | uniport | 0.175 | 0.284 | 0.333 | 0.301 |
|  | spscope | 0.115 | 0.182 | 0.202 | 0.196 |
|  | diffusion | 0.476 | 0.579 | 0.578 | 0.585 |
|  | SpaLSTF | 0.468 | 0.569 | 0.575 | 0.575 |
